# Supplementary material for: Risk factors associated with student distress in medical school: Associations with faculty support and availability of wellbeing resources
Source: PLoS One. 2022 Apr 8;17(4):e0265869. doi: 10.1371/journal.pone.0265869 (PMC8992977; doi:10.1371/journal.pone.0265869)
Supplement: Attachment — Submitted filename: Response to Reviewers.v2.docx [file pone.0265869.s003.docx]

- A rebuttal letter that responds to each point raised by the academic editor and reviewer(s). You should upload this letter as a separate file labeled 'Response to Reviewers'.
- A marked-up copy of your manuscript that highlights changes made to the original version. You should upload this as a separate file labeled 'Revised Manuscript with Track Changes'.
- An unmarked version of your revised paper without tracked changes. You should upload this as a separate file labeled 'Manuscript'.

1. Title: Suggest to change “Medical Schools Contribute to Student Distress: The Impact of Faculty Support and Wellbeing Resource Availability” to “Risk factors associated with student distress in medical schools: Associations with faculty support and wellbeing resource availability” **Edited as suggested.**
2. Line 50: Suggest to change to “Schools can help mitigate medical student distress by improving faculty support and offering more and varied wellbeing resources” to “Improving faculty support and offering more and varied wellbeing resources may help to mitigate medical student distress.” **Edited as suggested.**
3. Line 109: Suggest to change “…determine the impact of institutional investment in wellbeing resources on medical student wellbeing…” to “…determine whether there are associations between institutional investment in wellbeing resources and medical student wellbeing…” **Edited as suggested.**
4. Line 345-359: The first paragraph of the discussion needs to be rephrased to avoid using language implying causation, e.g. “we found that medical schools both contribute to and help mitigate against severe distress”. **Added “may” to avoid implying causation.**
5. Line 362: Suggest to change “In our study, having more wellbeing resources at one’s school was protective against severe distress. Interestingly, the availability of multiple wellbeing resources mitigated severe distress regardless of whether students utilized the resource.” to “In our study, having more wellbeing resources at one’s school was associated with less severe distress. Interestingly, the availability of multiple wellbeing resources moderated the relationship with severe distress regardless of whether students utilized the resource.” **Edited as suggested.**
6. Line 477-483: Revise conclusion to avoid language implying causation, e.g. “medical schools themselves play a role in medical student wellbeing” **Added “may” to avoid implying causation.**
